# Parental Alexithymia and Family Functioning in Families of Children with Autism Spectrum Disorder

**DOI:** 10.1192/j.eurpsy.2026.11170

**Published:** 2026-07-31

**Authors:** R. Kosić, D. Petrić, M. N. Bogdanovic, T. Kosec, R. Bajić

**Affiliations:** CHC Rijeka, Rijeka, Croatia

## Abstract

**Introduction:**

Alexithymia is characterized by an impaired ability to be aware of, explicitly identify, and describe one’s feelings. It was observed that a number of parents of children with autism spectrum disorder (ASD) exhibited traits resembling characteristics of ASD themselves, including difficulties in social interaction, specific patterns of cognitive functioning, and challenges in recognizing and expressing emotions.

**Objectives:**

The aim of this study was to examine the relationship between parental alexithymia and family functioning in families of children with autism spectrum disorder (ASD) compared to families of typically developing (TD) children.

**Methods:**

The study included 120 parents of children with ASD and 120 parents of TD children. Standardized instruments were used to assess alexithymia, parental stress, family cohesion and adaptability, emotional regulation strategies, resilience, and social support.

**Results:**

The results showed that parents of children with ASD had higher levels of alexithymia, greater parental stress, lower social support, and more frequent use of negative coping strategies. Alexithymia was significantly associated with poorer family cohesion and adaptability, regardless of stress or resilience levels. Key predictors of better family functioning included lower stress levels, higher social support, and positive emotional regulation strategies.

**Conclusions:**

These findings highlight the importance of interventions that promote emotional awareness and communication. These interventions can reduce parental stress, improve family functioning, and positively impact the child’s development.

**Disclosure of Interest:**

None Declared

